# Simultaneous *Viscum* pleurodesis and video-assisted thoracic surgery (VATS) bullectomy in patients with primary spontaneous pneumothorax

**DOI:** 10.1038/s41598-021-02224-z

**Published:** 2021-11-25

**Authors:** Hee Suk Jung, Hyun Jung Kim

**Affiliations:** Department of Thoracic and Cardiovascular Surgery, CHA Bundang Medical Center, CHA University, 59, Yatap-ro, Bundang-gu, Seongnam-si, 13496 Korea

**Keywords:** Diseases, Medical research

## Abstract

Although surgery is the gold standard for treatment of primary spontaneous pneumothorax (PSP), recurrence after surgery remains a concern. This study sought to evaluate the efficacy of simultaneous pleurodesis using *Viscum album* (VA) extract and video-assisted thoracic surgery (VATS) bullectomy for the treatment of PSP. From March 2016 to June 2020, 175 patients with PSP underwent bullectomy and intraoperative pleurodesis with VA extract at a single institution. All operations were performed through thoracoscopy by one surgeon. Upon completion of bullectomy, a polyglycolic acid sheet was used to cover the stapler lines, and 40 mg of VA extract was instilled over the entire chest wall before chest tube placement. The median operating time was 20 min (interquartile ranges, 15–30) and the median indwelling time of chest drainage was 2 days (interquartile ranges, 2–3). There were no postoperative complications over grade 3. During the median follow-up period of 38 months (interquartile ranges, 15–48), no recurrence of pneumothorax was observed. The results of this study demonstrated that simultaneous *Viscum* pleurodesis and VATS bullectomy provides a feasible and effective treatment option for preventing postoperative pneumothorax in patients with PSP.

## Introduction

The causes of primary spontaneous pneumothorax (PSP) have not been established, but it frequently arises in thin and tall male adolescents with no relevant medical history. In asymptomatic cases with lower pneumothorax volumes, an absence of air leakage, and no further lungs expansion, patients are treated conservatively with oxygen therapy. Closed thoracostomy and needle aspiration are the most widely used treatment method for pneumothorax. Tube thoracostomy not only expands collapsed lungs with continuous air leakage but can also be connected to a suction unit to increase the effectiveness of treatment. Needle aspiration is considered to be as effective and safe in patients with a first episode of small pneumothorax.

Non-surgical treatment can reverse pneumothorax but typically does not prevent recurrence. The estimated recurrence rate after the first episode of PSP is broad, ranging from 0 to 60 percent. One of the largest epidemiologic studies suggests rates of recurrent PSP were 20% in males and 22% in females over 5 years of follow-up^1^. In another systemic review, overall recurrence rate for patients with PSP was 32%^2^. Bullectomy should be considered in patients with recurrent pneumothorax. However, postoperative recurrence is relatively common after bullectomy^3–6^. Thus, several additional procedures including the use of fibrin sealants, radiofrequency therapy, and simultaneous pleurectomy or pleurodesis with bullectomy have been described to minimize the recurrence of PSP^5,7–10^.

In the present study, we evaluated the efficacy of intraoperative chemical pleurodesis using *Viscum album* (VA) extract after video-assisted thoracic surgery (VATS) bullectomy for the treatment of PSP.

## Methods

### Study design and patients

**Figure 1:**
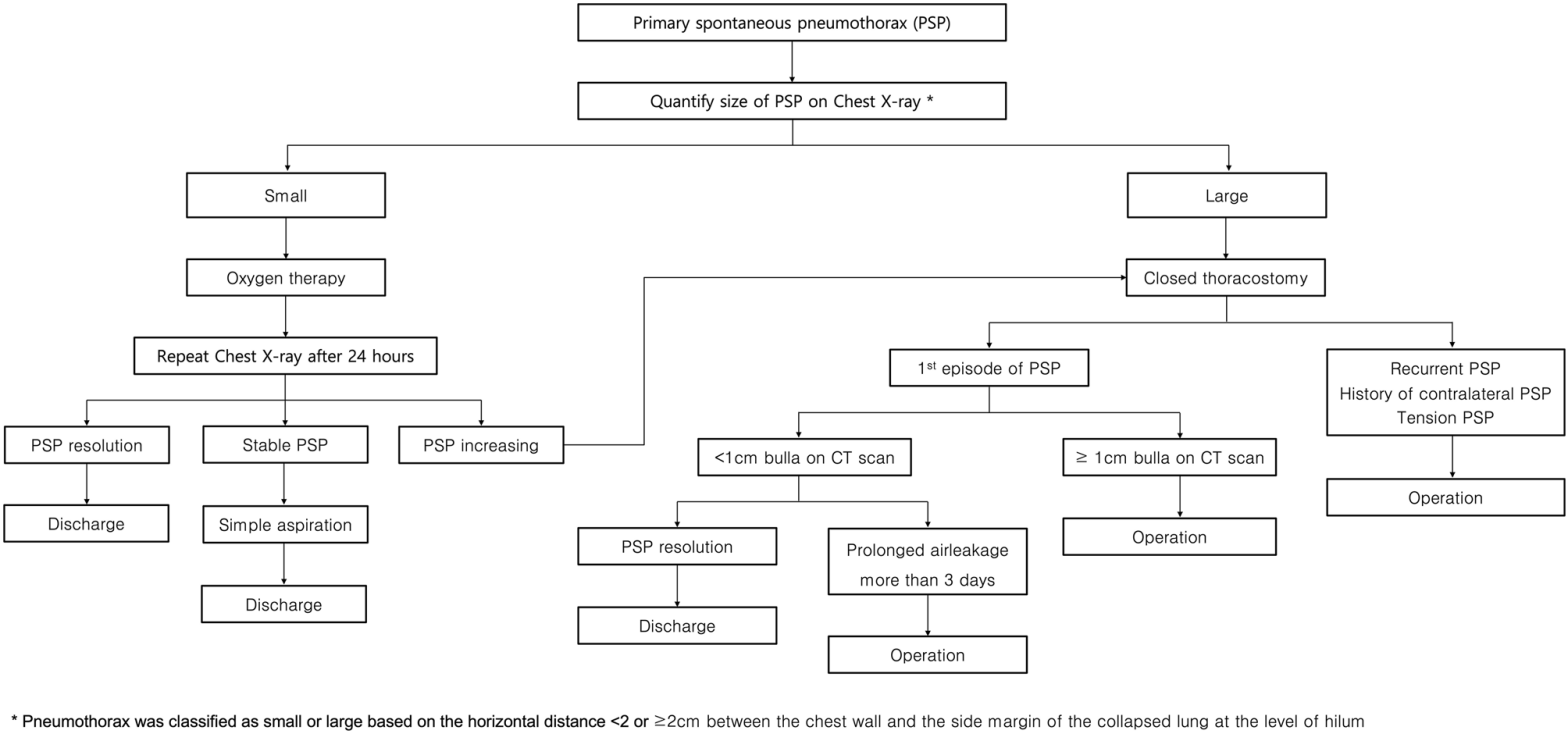
Treatment algorithm of primary spontaneous pneumothorax.

Between March 2016 and June 2020, 175 patients underwent VATS bullectomy and intraoperative pleurodesis using VA extract for PSP at our hospital. All patients were diagnosed with pneumothorax on chest radiography. The patients with a large amount of pneumothorax underwent chest computed tomography (CT) scans after closed thoracostomy. Figure 1 shows the treatment strategy for PSP used in our institution. Patients who had catamenial pneumothorax (related to menstruation), traumatic pneumothorax, or underlying lung disease (secondary pneumothorax) were excluded from the study. The eligibility criteria for surgery were as follows: recurrent pneumothorax, pneumothorax on the opposite side (contralateral pneumothorax), bilateral pneumothorax, tension pneumothorax, bulla or blebs over 1 cm on chest CT, air leakage for more than 3 days, and special occupations (e.g., pilots or divers). Postoperative recurrent pneumothorax was defined as a pneumothorax confirmed by chest radiography or CT scan that occurred 30 days or more after removal of the chest tube. The protocol was approved by the Institutional Review Board of Bundang CHA Medical Center (No. 2021-06-038), and the requirement for informed consent was waived due to the retrospective nature of the study. All procedures performed in this study were in accordance with the ethical standards of the national research committee.

### Procedures

**Figure 2:**
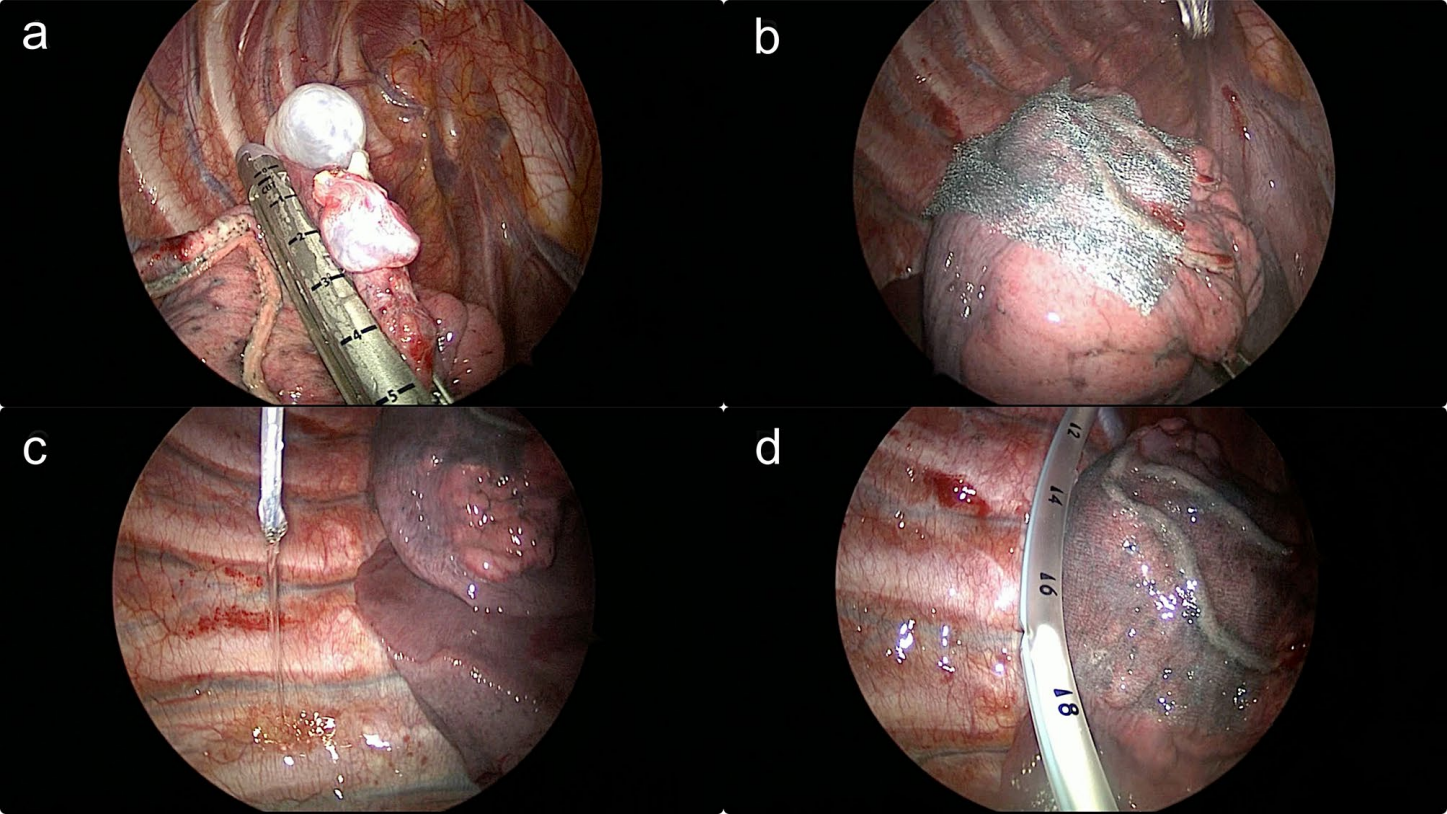
(**a**) Resection of bulla using an autostapler; (**b**) stapler line reinforcement with a polyglycolic acid sheet; (**c**) administration of a mixture of 40 mg VA extract and saline into the pleural space by needle instillation; (**d**) chest tube placement before lung inflation.

**Figure 3:**
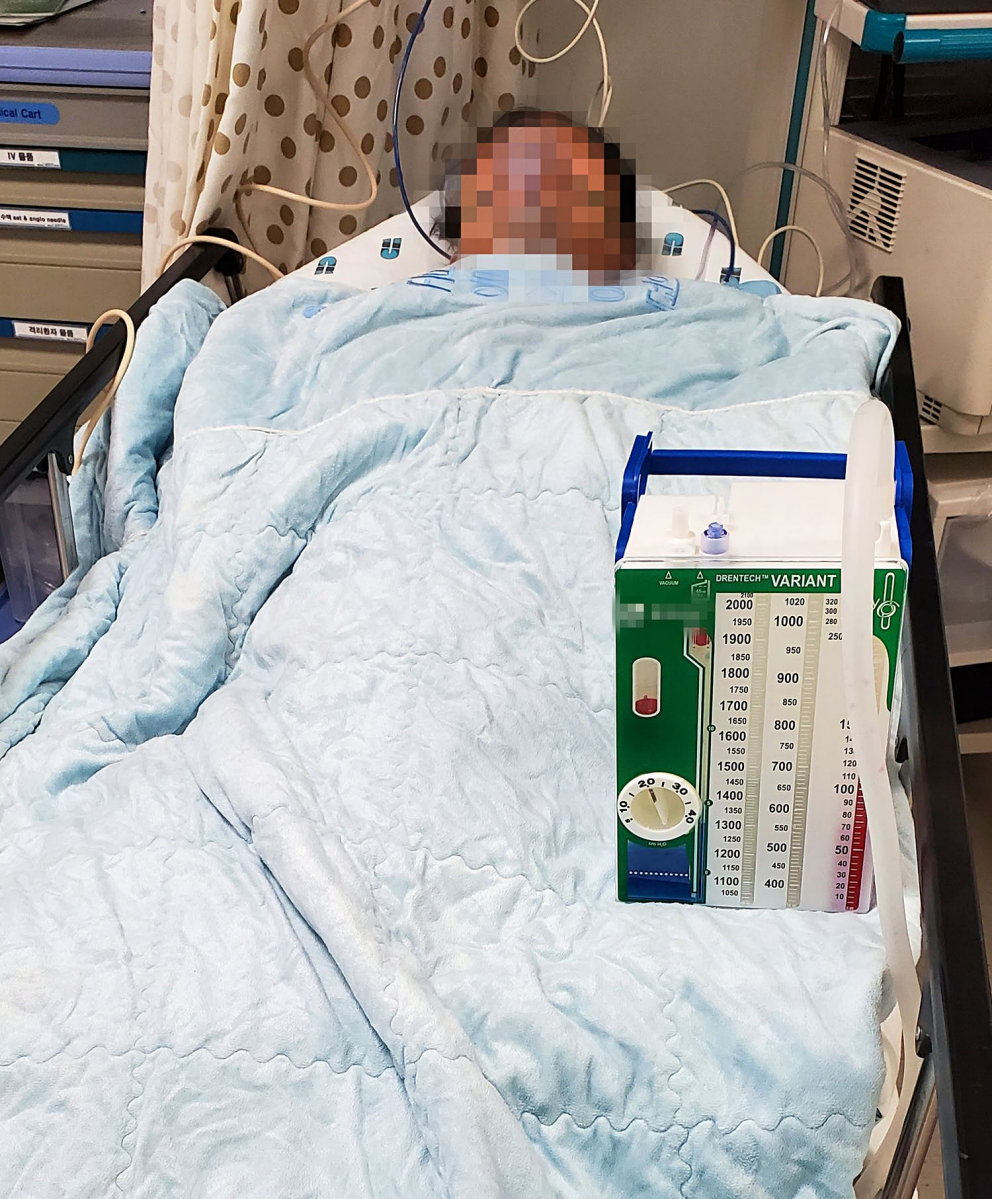
After the operation, the chest tube was lifted approximately 30 cm above the patient for 30 min without clamping in the recovery room.

All operations were performed by a single surgeon. Patients were placed under general anesthesia in the lateral decubitus position with a double lumen endotracheal tube. A camera port was inserted through the seventh intercostal space (ICS) along the mid-axillary line after lung deflation. Two working ports were inserted, one over the fifth ICS anterior axillary line and the other over the fourth ICS posterior axillary line. Any bulla or blebs identified on 3-mm 30° thoracoscope were resected with an endoscopic stapling device (EndoGIA, Auto Suture Company, Norwalk, CT, USA). After bullectomy, the stapling sites were covered with an absorbable polyglycolic acid sheet (Neoveil; Gunze Ltd., Kyoto, Japan) and fibrin glue. A mixture of 40 mg of VA extract (European Mistletoe, ABNOBA viscum F; ABNOBA Helmittel GmbH, Pforzheim, Germany) and 50 mL of normal saline was administered into the pleural space by needle instillation (Supplementary Video 1; Fig. 2). Care was taken to ensure that the mixed agent was sprayed as directly towards the parietal pleura and evenly applied to the entire chest wall and surface of the diaphragm. All ports were removed under direct vision and a 20-Fr chest tube was inserted through the port sites followed by lung inflation. After surgery, the connected tube line was lifted up approximately 30 cm above the patient for 30 minto avoid outflow of the sclerosing agent, allowing air to pass (Fig. 3). After drainage of the agent, the chest tube was connected to a low-pressure suction system at − 10 cm H_2_O.

### Postoperative care and follow-up

Postoperative analgesics included routine oral non-steroid analgesics and intravenous injection of pethidine hydrochloride if the pain became intolerable. Chest pain or discomfort was recorded using a numeric rating scale (NRS) from 0 to 10; severe pain was defined as a NRS score of 7 or more. Chest radiography was taken immediately postoperatively and every morning. The criterion for removal of the chest tube was full expansion of the lung on chest radiography with cessation of air leakage. After discharge from the hospital, patients were monitored at the outpatient clinic 2 weeks, 1 month, and 6 months postoperative via chest radiography and physical examination. Patients who had passed the 6-month follow-up period were asked to report the recurrence of pneumothorax by telephone questionnaire once a year.

## Results

The median age of the 175 patients who underwent surgery for PSP was 20 years old (interquartile ranges, 15–40), and males accounted for 97.1%. In total, 34 (19.4%) out of the 175 patients underwent treatment for recurrent pneumothorax; 10 who had received conservative treatment with oxygen therapy; 18, closed thoracostomy; 6 who underwent bullectomy and were transferred to our hospital for recurrent pneumothorax. There was no difference in the incidence rate of PSP on the left and right side. The median interval from first operation for PSP was 6 months (interquartile ranges, 2–8) for ipsilateral recurrent pneumothorax and 13 months (interquartile ranges, 10–18) for contralateral pneumothorax, respectively (Table 1). Median operating time was 20 min (interquartile ranges, 15–30) and median indwelling time of chest drainage was 2 days (interquartile ranges, 2–3). There were no postoperative complications including bleeding, atelectasis, pneumonia, arrhythmias, or wound infections. During the median 38 months (interquartile ranges, 15–48) follow-up period, no recurrences or symptoms that suggested pneumothorax were observed (Table 2).

**Table 1:** Clinical demographics of the study subjects.

Characteristics	Total (n = 175)
Age (years) at operation	20 (17–32)
Sex (male:female)	170 (97.1) : 5 (2.9)
**Affected side**	
Right	63 (36.0)
Left	100 (57.1)
Bilateral	12 (6.9)
Tx history for ipsilateral pneumothorax	34 (19.4)
Observation	10 (5.7)
Closed thoracostomy	18 (10.3)
Operation	6 (3.4)

Data are presented as No. (%) or median (interquartile ranges).

*Tx* treatment.

**Table 2:** Surgical and postoperative results.

Outcomes	Value
Operation time (min)	20 (15–30)
No. of bullectomies	3 (2–4)
**Body temperature**	
POD #0/POD #1	37.4 (37.2–38.0)/37.2 (36.8–37.6)
**Pain scale (NRS)**	
POD #0/POD #1	4 (3–5)/3 (2–3)
Postoperative chest tube drainage (days)	2 (2–3)
Postoperative hospital stay (days)	2 (2–4)
Postoperative complications (grade ≥ 3)	0 (0)
Recurrence	0 (0)
Follow-up period (months)	38 (15–48)

Data are presented as No. (%) or median (interquartile ranges).

*POD* postoperative day, *NRS* numerical rating scale.

## Discussion

One of the most important issues in the treatment of PSP is preventing recurrence. Although VATS is the most effective treatment for PSP, the postoperative recurrence still remains after bullectomy alone with rates ranging from 6 to 27.9%^3–6,11–13^. In order to minimize recurrence rate of postoperative pneumothorax, chemical pleurodesis has been performed simultaneously with resection or sequentially. It can significantly decrease the risk of early air-leakage or late recurrence. Chen et al. reported a significant reduction in pneumothorax recurrence in a group who received additional minocycline pleurodesis after bullectomy (2%) compared with those who only underwent bullectomy (8%)^9^. A similarly reduced recurrence rate was also demonstrated by Loubani et al. who showed that intraoperative tetracycline pleurodesis following surgery appears to have less risk of ipsilateral recurrence, but longer postoperative stay^10^.

Sterile talc without asbestos is the most widely used agent in chemical pleurodesis. However, this agent should be used carefully and only in selected patients because it can cause serious complications such as acute respiratory distress syndrome, systemic embolism, and acute pneumonitis. Other sclerosing agents include a derivative of tetracycline, autologous blood, povidone iodine, OK-432, etc., but these are associated with higher rates of recurrence of pneumothorax than sterile talc^14–19^.

VA extract is one of several sclerosing agents under investigation for their potential ability to increase efficacy and fewer complications^20^. This extract is widely used in the treatment of many diseases in traditional and complimentary medicine due to its anti-tumor effects and immune modulation^21^. VA extract was first used by Salzer et al. in 1977 for chemical pleurodesis in the treatment of malignant pleural effusion to stimulate antitumor immunity rather than mechanical sclerosis^22^. Cho et al. reported that chemical pleurodesis using VA extract was a successful means of treating malignant pleural effusion in 79% (49/62) of patients^23^. This drug is also used as an effective sclerosing agent in cases of congenital chylothorax and pneumothorax with continuous airleakage^24–28^. The complications of *Viscum* pleurodesis that have been reported are mostly minor and include transient pleural effusion, a mild burning sensation, and mild fever episodes; there have been no reports of serious complications.

**Figure 4:**
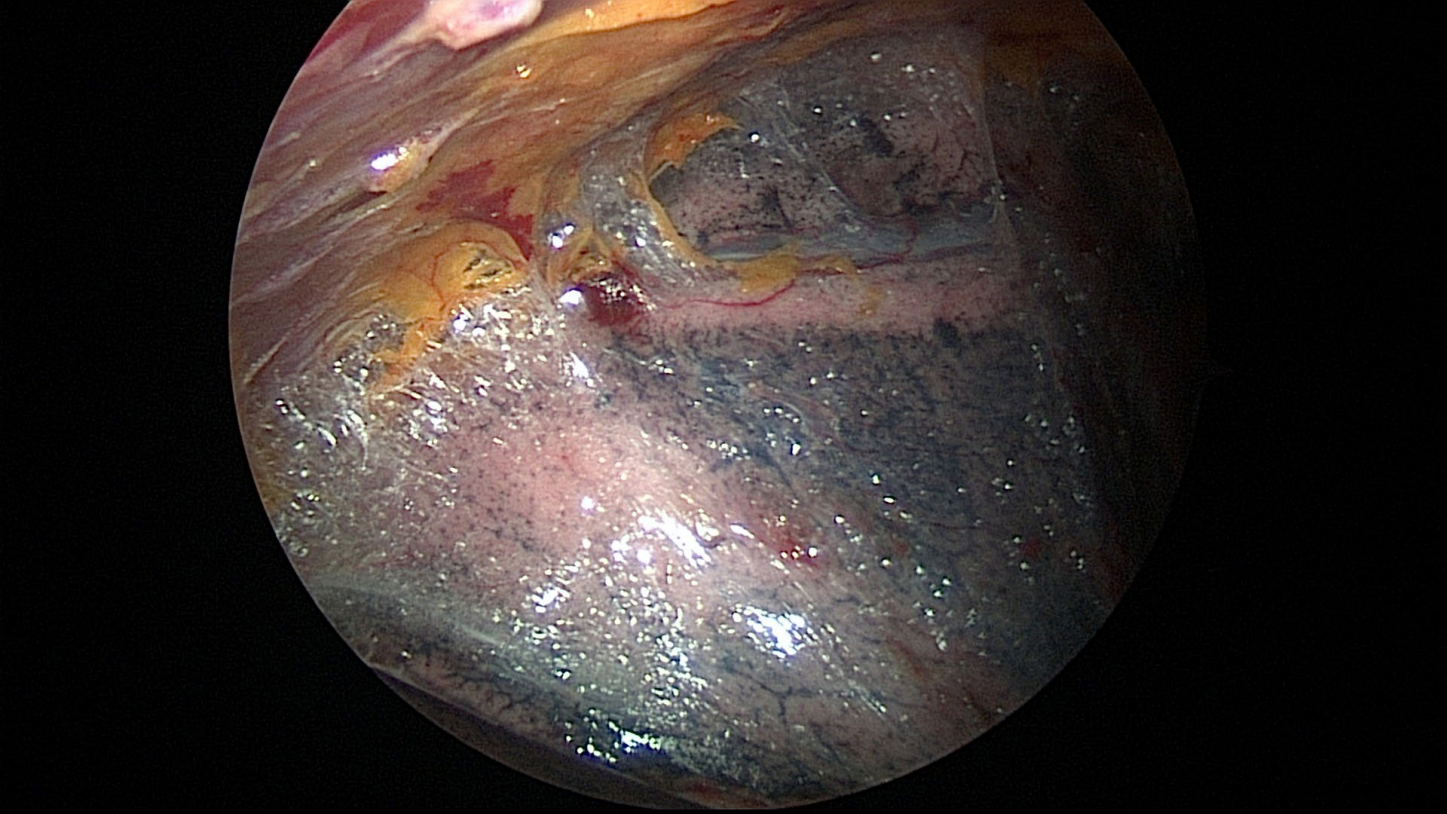
Intraoperative photograph of the flimsy pleural adhesions after simultaneous *Viscum* pleurodesis and bullectomy.

Although chemical pleurodesis is an additional technique to prevent the recurrence of postoperative pneumothorax, it has several side effects, such as aggravated postoperative pain due to pleural irritation or inflammation. Moreover, there is a possibility of restrictive pulmonary dysfunction caused by pleural adhesions which can make repeat pleurolysis challenging. However, several papers reported that pain after pleurodesis was well controlled via patient-controlled-analgesia or narcotic analgesics, and patients did not exhibit a significant degree of pulmonary hypofunction compared to those in the group free from pleurodesis^5,10^. In a patient undergoing VATS excision for a thymic cyst 6 months after PSP surgery in our study, there was no difficulty performing pleurolysis with dissection via electrocautery due to scant and loose adhesions between the visceral and parietal pleura (Fig. 4). We used 40 mg of VA extract, which was approximately one-third of the dose for malignant effusion. Besides, conventional pleurodesis requires 4 to 6 h of indwelling time, whereas VA extract was drained out 30 min after surgery in the recovery room. This dosage and indwelling time for *Viscum* pleurodesis with bullectomy were effective for preventing pneumothorax recurrence without serious complications; however, further research is needed to confirm our findings.

This is an uncontrolled, single center retrospective report, and without a control group to evaluate this agent against other sclerosing agents (e.g. doxycycline, talc, iodine, bleomycin, fluorouracil, silver nitrate etc.). This field requires a multi-center comparative study to sort out the agent with the least toxicity, adverse effect, and greatest long-term efficacy.

## Conclusion

Surgical resection alone is not sufficient for preventing recurrence of pneumothorax. A comparative study with other sclerosants is needed to determine the efficacy of VA extract in pleurodesis, but this study showed good results with less morbidity. We suggest that simultaneous *Viscum* pleurodesis and VATS bullectomy could be a feasible and effective treatment option for patients with PSP.

## Supplementary Information

Supplementary Legends.

Supplementary Video 1.

## Abbreviations

CT: Computed tomography
ICS: Intercostal space
NRS: Numeric rating scale
POD: Postoperative day
PSP: Primary spontaneous pneumothorax
Tx: Treatment
VA: *Viscum album*
VATS: Video-assisted thoracic surgery
