# Supplementary material for: Simultaneous Viscum pleurodesis and video-assisted thoracic surgery (VATS) bullectomy in patients with primary spontaneous pneumothorax
Source: Sci Rep. 2021 Nov 25;11:22934. doi: 10.1038/s41598-021-02224-z (PMC8617264; doi:10.1038/s41598-021-02224-z)
Supplement: Supplementary file 1 — Supplementary Legends. [file 41598_2021_2224_MOESM1_ESM.pdf]

Video 1. Intraoperative view of bullectomy followed by *Viscum* pleurodesis.
